# Burnout and work-privacy conflict – are there differences between full-time and part-time physicians?

**DOI:** 10.1186/s12913-022-08471-8

**Published:** 2022-08-24

**Authors:** E Bodendieck, FU Jung, M Luppa, SG Riedel-Heller

**Affiliations:** 1 General Practice, Dresdner Straße 34a, 04808 Wurzen, Germany; 2 Institute of Social Medicine, Occupational Health and Public Health, Medical Faculty, University of Leipzig, Ph.-Rosenthal-Str. 55, 04103 Leipzig, Germany

**Keywords:** Physician, Part-time, Work-privacy conflict, Burnout

## Abstract

**Background:**

Changes in everyday work with regard to working time models have reached the medical profession. The number of physicians working part-time is steadily increasing. At the same time, however, the population's need for care is also rising. This can reinforce the impending shortage of doctors in the future. The aim was to investigate differences in work-privacy conflict and burnout among physicians working full-time or part-time.

**Method:**

The present study includes data from a baseline survey of the long-term study of physicians with different medical backgrounds. The analysis focused on a sub-sample of 598 physicians (not self-employed). The two main outcomes under investigation—burnout and work-privacy conflict—were measured using the Copenhagen Burnout Inventory adapted for health care professionals, as well as the associated subscale of the Copenhagen Psychosocial Questionnaire (COPSOQ). Data analyses included descriptive statistics followed by regression models.

**Results:**

Descriptive analyses show, that 31.8% of physicians are working part-time, whereas 68.2% are working full-time. The part-time subsample is significantly older, and female physicians are more likely to work part-time. With regard to workload and work-privacy conflict, significant differences between part-time and full-time physicians were only observed in terms of work-privacy-conflict. However, regression analysis underline the importance of possible confounding variables (such as medical setting) within the relationship between job size and job-related well-being.

**Discussion:**

Differences in working hour arrangements (full-time or part-time work) are only accompanied by less work-privacy conflict. No differences with regard to burnout (patient-related, work-related or personal) could be obtained. Rather, the data suggests that other job-related variables may play a role and should be further investigated.

## Introduction

Physician shortage has been one of the major strains in health care worldwide and can be found in inpatient as well as outpatient settings. Often, turnover rate is related to permanent workload and dissatisfaction with occupational conditions [1, 2]. In a sample containing clinical physicians, working excessively long hours was found in every fifth physician [3]. In this context, working excessively long hours was related to more health complaints, including mental and physical fatigue. In order to change this development and maintain patient care on a sufficient level, it has been argued that flexible working time arrangements may provide security, psychological compensation and relief in order to decrease negative consequences of workload [4–6]. Currently, physicians – especially younger generations – wish for more flexibility, as well as the possibility to work part-time [7].

However, part-time work is often related to personal and professional reasons, for instance working as a surgeon is associated with full-time work, whereas having children is associated with part-time work [8]. The desire for part-time work may be found more often in female physicians and younger professionals. A recent study with a focus on part-time physicians [9], demonstrates that physicians working part-time are more likely to prefer working in private practices compared to their full time working colleagues.

When talking about consequences of part-time work, previous research often tries to shed light on both sides – physician’s wellbeing in terms of workload, risk for burnout and stress, and on the other hand satisfaction with work-life balance. Overtime work as well as high number of hours worked per week has been shown to increase workload and the risk for developing burnout [1]. Flexible arrangements with regard to working time may reduce burnout risk, and improve overall job satisfaction, exhibited by physicians working within hospital settings [10]. In addition, studies that focus on physicians working in outpatient care also show that part-time work is associated with less burnout and more satisfaction [6]. On the other hand, it has been suggested that part-time work may be associated with greater work density [11] and that working time reduction per se may not reduce the risk of developing burnout [12]. The question arises whether part-time physicians exhibit higher levels of burnout risk due to time restrictions, or lower levels because they may have more time for recovery. Therefore, results are still inconsistent and need further investigation.

When it comes to improving working conditions (and hence reducing burnout), it has been suggested that work-family or work-privacy enrichment plays an important role [13]. In general, physicians have been shown to exhibit more work-family conflict (WIF) compared to the general population [15]. According to previous research, it has been known that higher work privacy or work family conflict may correlate with stress and burnout [14, 15], as indicated by greater risk for burnout in physicians caring for younger children [16]. However, in a recent study comparing physicians working in general medicine, living in a household with children under the age of 18 did not significantly affect work-privacy conflict [15]. Besides, work-privacy conflict may also arise if physicians do not have the time for training, academic or volunteer professional work they would like to have.

According to the literature, there may be differences between full-time and part-time employees, as part-time working physicians (compared to full-time or overtime working physicians) seem to be more satisfied with the time they are able to allocate to their private and family life [17, 15]. Similarly, another study suggests that job size is negatively related to satisfaction with the balance between work and private hours [18]. In other words, small job size was related to a greater satisfaction with this balance. However, this aspect was only measured using a single item and no information is available with regard to medical background (e.g. whether physicians provide ambulatory or inpatient care).

In summary, we aim to investigate differences between full-time and part-time physicians in a broad sample of physicians with different medical backgrounds. So far, there is no strict regulation or definition of full- or part-time work with regard to an exact number of working hours [19] and even within the medical profession in Germany, there is a range between 38.5 and 48 h per week, that has been defined as full-time [20]. In Germany, full-time work is most commonly based on a eight-hour-day and 40 h per week. Therefore, we define full-time work by 40 h per week or more and part-time work by less than 40 h per week, as suggested by a similar study investigating satisfaction and burnout in part-time physicians [6].

Especially since legal regulation with regard to working hours have been changed recently, it is important to further examine the status quo and determine how this may have changed physicians’ (occupational) well-being. Therefore, the aim of this study was to compare part-time and full-time physicians in terms of (self-reported) burnout, as well as work-privacy conflict. This will include analyses with regard to the medical setting (outpatient vs inpatient), as well as geographical information (physicians working in rural or urban places) and socio-demographic information (family status, age, gender and the presence of younger children).

## Method

### Study design and sampling procedure

The data under analysis were taken from a long-term study of physicians working in the Federal State of Saxony (Germany). We analyzed data from the baseline survey, which was conducted in February 2020 and includes 1,001 physicians (response rate: 33.4%). Physicians were randomly selected out of a pool of registered physicians in Saxony. They were asked to fill in and return a questionnaire by post. All information collected by the questionnaire has been pseudonymized for longitudinal use. The final sample used for statistical analysis consists of physicians currently working in direct patient care – either in hospital or in ambulatory care, and who are not self-employed. As retirement status could influence the results, we excluded physicians older than 66 of age from the analysis. Informed consent has been obtained from all participants. All methods were performed in accordance with the relevant guidelines and regulations.

### Assessment

In addition to socio-demographic (e.g. age and gender) and job-specific aspects (e.g. hospital or ambulatory care), questions about working hours were part of this survey. Participating physicians were asked about their average number of hours per week (as agreed by contract, median in this sample: 40 h/week). Physicians were then categorized as working part-time (less than 40 h per week) or working full-time (40 h per week or more) as has been suggested before [6]. In order to determine whether physicians are working in rural areas or large cities, they were asked the following question (predefined categories): “How many people live in the region of your workplace?”. Physicians in this sample were also asked whether they do overtime hours on a regular basis (yes or no).

Burnout was investigated using the Copenhagen Burnout Inventory [21], that consists of 19 items that need to be answered on a five-point scale. The CBI-scale was adapted for professionals working in health care [22] and includes three subscales: personal burnout (e.g. “How often are you physically exhausted?”, 100 = always/ 0 = never), work-related burnout (e.g. “Do you feel burnt out because of your work?, 100 = to a very high degree/ 0 = to a very low degree) and patient-related burnout (e.g. “Do you find it hard to work with patients?”, 100 = to a very high degree/ 0 = to a very low degree). In order to compare the score to findings from the literature, an overall burnout score containing three items from the personal burnout subscale was calculated, as described elsewhere [23]. The Cronbach’s alpha for this score is 0.81.

Work-privacy conflict was investigated using a scale developed by Nübling [24, 25]. The scale is part of the Copenhagen Psychosocial Questionnaire (COPSOQ). A current version can be downloaded online [26]. For the purpose of this study, the German version that was available online at the time of the data collection in 2019, has been used. The Cronbach’s α for the overall scale containing seven items is 0.85. Completion rates of the overall scale were high (97.7%—98.7%). Item 7 does not fit well in the scale due to very low item-test and item-rest correlations (< 0.5, [27]) and was therefore excluded from analysis, leaving a Cronbach’s α of 0.89. In addition, an explorative factor analysis (principal component analysis with varimax rotation) was conducted using the remaining six items. Table 1 gives a summary of the scale analysis and the items of the scale. Based on the visual analysis of the scree-plot and interpretation of further empirical factors, two dimensions of work-privacy conflict were identified (Factor 1 and Factor 2, see Table 1).

**Table 1:** Summary of scale analysis (work-privacy conflict scale)

Item	Item-test correlation	Factor loadings	
		Factor 1	Factor 2
1. The demands of my work interfere with my private and family life	0.84	0.84	
2. The time invested in my job makes it difficult for me to carry out my family or private life responsibilities	0.86	0.88	
3. My work drains so much of my energy that it has a negative effect on my private life	0.81	0.82	
4. My work takes so much of my time that it has a negative effect on my private life	0.86	0.89	
5. There are times when I am supposed to be at home and at work at the same time	0.77	0.62	
6. I also do professional things outside my working hours	0.58		0.75

Note: response categories: to a very large extent / to a large extent / some – what / to a small extent / to a very small extent

### Data analysis

STATA 13 SE statistical software was used for all statistical analyses. In addition to descriptive data analysis (independent t-tests for continuous variables and Chi^2^-tests for categorical variables), regression analyses were conducted, assuming a significance level of 0.05. The independent variable is job size (dichotomized as part-time vs. full-time and as a continuous variable). The dependent variables include overall burnout (Model 1), patient-related burnout (Model 2), work-related burnout (Model 3), personal burnout (Model 4) and work-privacy conflict (Model 5).

Size of region was calculated from the number of inhabitants stated by the physician and categorized into three groups: small (less than 20,000 inhabitants), middle (20,001–50,000 inhabitants), large (more than 50,001 inhabitants).

Cases with missing information were omitted from final analyses.

## Results

The majority in the overall sample was female (*n* = 371, 62.0%) and on average 41.9 years old (standard deviation: 11.1, range: 25–66). With regard to the medical setting, 469 physicians (78.4%) were working in a hospital and 44.3% of physicians stated working in areas with more than 500,000 inhabitants. Overall, 31.8% can be categorized as part-time and 68.2% as full-time employees. Out of the 469 hospital-physicians, 27.1% were working part-time. Contrary, out of the 131 physicians working in ambulatory care, 48.8% were working part-time.

The sociodemographic details with regard to the two study groups (part-time vs. full-time) are given in Table 2. The sample containing part-time physicians was significantly older (*p* < 0.001) and more physicians in this sub-sample were female compared to the sample containing full-time physicians (*p* < 0.001). The average score for burnout was 56.2 (burnout: patient related = 22.4; work-related = 36.8; personal = 45.8) and 45.8 for work-privacy-conflict. Overall, there was no significant difference in overall burnout between part-time and full-time physicians (p = 0.343), but a significant difference with regard to work-privacy-conflict (*p* < 0.001).

**Table 2:** Sociodemographic characteristics of the study population (*n* = 598)

	Part-time physicians (n = 190)	Full-time physicians (n = 408)
Age***	Ø 44.0	Ø 41.0
Gender (female)***	155 (81.6%)	216 (52.9%)
Marital status *		
married	126 (66.3%)	224 (54.9%)
In a relationship	38 (20.0%)	107 (26.2%)
single	26 (13.7%)	77 (18.9%)
Children under the age of 14 (yes)***	113 (59.5%)	162 (39.7%)
Medical Setting***		
inpatient	127 (66.8%)	342 (83.8%)
outpatient	63 (33.2%)	66 (16.2%)
Number of inhabitants near the workplace, n.s		
< 20,000	58 (30.5%)	97 (23.8%)
20,001 – 500,000	55 (29.0%)	123 (30.1%)
> 500,001	77 (40.5%)	188 (46.1%)
Overtime hours (yes)***	112 (58.9%)	300 (73.5%)
CBI – burnout		
burnout score, n.s	Ø 56.8	Ø 55.9
patient related, n.s	Ø 21.5	Ø 22.9
work-related, n.s	Ø 37.0	Ø 36.8
personal, n.s	Ø 45.9	Ø 45.7
COPSOQ—Work-Privacy Conflict***	Ø 39.2	Ø 48.9

Note: *CBI* Copenhagen burnout inventory, *COPSOQ* Copenhagen psychosocial questionnaire; n.s. = not significant, * = *p* < 0.05; ** = *p* < 0.01; *** = *p* < 0.001

In addition, multiple regression analysis was conducted in order to investigate the association between work hours (independent variable, dichotomized) and burnout score (model 1), all three dimensions of burnout (model 2, 3 and 4) as well as work-privacy-conflict (model 5). The results are summarized in Table 3 (using working hour as a dichotomized variable) and Table 4 (using working hours as a continuous variable). Sociodemographic and work-related factors, such as age, gender, marital status, child care and medical setting (inpatient or outpatient), overwork and size of region with regard to the workplace were included as confounding variables.

**Table 3:** Prediction of job-related characteristics by work hours (part-time vs. full-time)

	Model 1 CBI Burnout risk	Model 2 CBI patient- related	Model 3 CBI personal	Model 4 CBI work-related	Model 5 COPSOQ – Work-privacy Conflict
Job Size (Part-time)^1^	-1.67	-0.42	-0.99	-0.09	7.28***
Age	-0.38***	-0.33***	-0.32***	-0.29***	-0.31***
Gender (male)	5.71**	-2.06	5.74***	4.38**	0.78
Marital status (married)					
In a relationship	-1.58	0.48	-1.24	-0.72	0.93
single	3.50	2.99	2.71	2.58	0.38
Children (no)	1.68	-3.30	0.21	-2.89	5.47**
Medical Setting (inpatient)	-2.95	5.44**	-1.94	-1.87	-5.67**
Size of region					
middle	0.38	-4.90*	-0.31	.2.12	0.29
large	-1.31	-7.05***	-3.48	-6.46***	-4.46*
Overtime hours (yes)	7.58***	4.95**	8.52***	8.50***	13.29***
Constant	64.64	38.62	52.22	52.53	44.57
R^2^	0.12	0.07	0.11		0.22

Note: ^1^ = dichotomized, full-time vs. part-time; * = *p* < 0.05; ** = *p* < 0.01; *** = *p* < 0.001

**Table 4:** Prediction of job-related characteristics by work hours (continuous)

	Model 1 CBI Burnout risk	Model 2 CBI patient- related	Model 3 CBI personal	Model 4 CBI work-related	Model 5 COPSOQ – Work-privacy Conflict
Work hours^1^	-0.02	-0.04	-0.02	-0.02	0.42***
Age	-0.37***	-0.33***	-0.32**	-0.29***	-0.30***
Gender (male)	6.08***	-2.08	5.92**	4.47**	0.27
Marital status (married)					
In a relationship	-1.57	0.49	-1.23	-0.72	0.87
single	3.44	3.02	2.69	2.54	0.20
Medical Setting (inpatient)	-2.81	5.39**	-1.88	-1.80	-5.44**
Children (no)	1.94	-3.28	0.35	-2.84	4.81**
Size of region					
middle	0.33	-4.90*	0.28	-2.12	0.41
large	-1.34	-7.05***	-3.50	-6.47***	-4.34*
Overtime hours (yes)	7.47***	4.97**	8.47***	8.64***	13.28***
Constant	63.43	39.92	52.00	42.68	33.90
R^2^	0.12	0.07	0.11	0.11	0.22

Note: ^1^ = continuous, full-time vs. part-time; * = *p* < 0.05; ** = *p* < 0.01; *** = *p* < 0.001

When comparing between full-time and part-time physicians, job size only significantly predicted work privacy conflict, but not burnout. Physicians working full-time reported more work-privacy conflict compared to their colleagues working part-time (Model 5). No relationship was found between working hour arrangement and either burnout (overall score, Model 1) or with regard to the three individual dimensions of burnout (Model 2–4).

Age was negatively associated with all constructs under investigation. In other words, younger physicians exhibited more burnout and more work privacy conflict. Gender was only positively associated with overall burnout, as well as work-related and personal burnout, so that female physicians in this sample exhibit more burnout. Marital status was not related to either burnout or work-privacy-conflict, whereas having children younger than 14 years of age was positively associated with work-privacy-conflict. Significant associations could also be found between medical setting (inpatient or outpatient) and patient-related burnout, as well as work privacy conflict. In other words, physicians working in an ambulatory setting exhibited more patient-related burnout, but less work-privacy conflict compared to their colleagues in hospital care. The size of the region with regard to number of residents did significantly affect patient- and work-related burnout. In other words, physicians working in areas with more than 20,000 residents exhibited less patient-related burnout and working in areas with more than 500,000 inhabitants accounted for less work-related burnout. In addition, working in areas with more than 500,000 inhabitants decreased work-privacy conflict.

In a second analysis step, working hours were included as a continuous variable (Table 4). Again, there was only a significant relationship between working hours and work-privacy conflict, but not burnout. All other results, with regard to confounding variables, did not reveal any differences with regard to significant associations and are therefore comparable to results from Table 3.

## Discussion

The aim of the study was to compare full-time and part-time physicians with regard to the level of burnout and work-privacy conflict. Overall, 31.8% of physicians in this sample are working less than 40 h per week. Previous studies for example reported 8.5% of part-time physicians in 2000 [17] and 16.5% in 2016 [28]. Therefore, there may be a trend within the medical profession to work part-time [29], as indicated by another study, showing that approximately 80% of (part-time and full-time) physicians wish to reduce their job size [18]. However, one should keep in mind that findings may differ between physicians working in hospitals and physicians providing ambulatory care, where the prevalence of part-time working doctors is often higher [30]. Additionally, women in our sample are more likely to work part-time, similar to other studies [31, 18, 6]. Interestingly, the part-time sub-sample is slightly, but significant older than the full-time sub-sample (44 vs 41 years of age). In this context, previous research suggests that working part-time may not be limited to younger physicians [29]. One possible explanation could be that senior physicians may want to reduce their workload, as the time between 10 and 20 years after entering the medical profession may be exceptionally stressful [32]. On the other hand, it may be difficult for younger physicians to find a part-time position when still receiving speciality training or professional education, because postgraduate training is mostly connected to full-time contracts [20], therefore explaining why younger physicians in our sample are more likely to work full-time despite increased levels of burnout and work-privacy conflict.

With regard to the overall aim, differences between full-time and part-time physicians can be summarized as follows. No significant differences between part-time and full-time physicians were obtained by comparing scores of burnout, similar to a study investigating burnout in a sample of Portuguese physicians [33]. Moreover, these results remain stable after introduction of control variables (such as age and gender). Multivariate regression analyses revealed no significant differences in terms of overall burnout or specifically for all three dimensions (patient-related, work-related or personal). Therefore, physicians working part- time do not exhibit higher scores in burnout compared to their colleagues working full-time. Similarly, including working hours as a continuous variable did not change any of these results. Therefore, results with regard to job size and job-related characteristics such as burnout and work-privacy conflict are relatively robust. These findings tie well with a previous study that concludes that working time reductions are not accompanied by reductions in work strain or risk of burnout in a sample of hospital physicians [12].

In greater detail, the results show, that being female was associated with greater work-related and personal burnout, as well as higher risk for burnout. No associations were found between gender and work-privacy conflict. In addition, being younger was associated with more burnout (overall). Family status did not reveal any significant differences, however, having children below the age of 14 does increase work-privacy conflict (but not burnout), which might explain why parental status positively influence the decision to work part-time in order to overcome the double burden of work and child care [8]. Due to methodological variations in previous studies (e.g. with regard to sample characteristics or region) it is difficult to compare the results. Most studies, that focus on work-life balance or work family conflict and parental status, only include female physicians. One study for example, investigating work- life balance in female gynaecologists has found that having fewer children at home did significantly predict work-life balance [34].On the other hand, there are studies that do not find any relationship between having children and burnout or work-family conflict [15, 28]. It may depend on the number of children and the possibility to make use of external childcare offers. In this context, offering the possibility to work part-time to female and male physicians or provide more on-site childcare might help to reduce work-family conflict and therefore attract or keep more physicians within healthcare. Interestingly, physicians working in more rural regions exhibit more burnout (patient and work-related burnout) according to regression analysis. Unfortunately, there are not many studies in Germany that investigate the relationship between region and level of burnout. In a previous study [35], we did not find any differences between rural and metropolitan regions, however, the sample of the preceding study was not comparable to the current sample in terms of sociodemographic characteristics (such as age and gender). A study that took place in Switzerland found a higher degree of burnout in physicians practicing in rural areas [36]. Burnout rates may be higher due to the length of commute time (between workplace and home), the lack of personnel or the higher number of patients that need to be treated due to physician shortage in rural areas. More research is needed to investigate the link between burnout and regional differences.

Overall, physicians exhibit higher levels of burnout compared to other studies. The burnout score was on average 56.2, compared to a study by Lincke et al., obtaining a score of 48.5. Patient-related burnout was lower (22.4 on average) compared to other studies (24.9–35.3, [37, 21, 38, 33]. Work-related burnout was similar (36.8 on average) compared to other findings (36.7–55.9, [21, 38, 33]. Scores obtained for personal burnout (45.8 on average) go align with what can be found in the literature (35.9–57.4, [21, 38, 33]. The differences in burnout could be explained by the consistency of the sample, for instance sociodemographic characteristics as well as job-related characteristics. Research investigating, whether different medical specialities may exhibit different levels of burnout has not revealed consistent results so far [1, 39, 40].

With regard to the second aim of this study, significant differences could be found in the context of work-privacy conflict, meaning that full-time physicians exhibit higher levels of conflict compared to part-time physicians. In-depth analysis shows that younger physicians in our sample exhibit more work-privacy-conflict. Another factor that influenced the size of conflict, was working in a hospital or inpatient setting. Compared to physicians in ambulatory care, hospital physicians exhibit more work-privacy conflict. This may be because working in a hospital means having less flexible working time arrangements. Additionally, the work may be characterized by more overwork compared to working in ambulatory care. This may be further explained by our data, as physicians working in hospitals are more likely to exceed the number of work hours as stated in their contract compared to physicians working in outpatient care. Interestingly, physicians working in areas with more than 500,000 inhabitants show less work-privacy conflict. So far, there is no study comparing urban and rural physicians in terms of work-life-balance, therefore more research is needed to further investigate this finding. This may help to make changes with regard to working conditions and hence attract more physicians to work in rural areas, combating physician shortage.

In general, compared to another study including physicians [41], the mean score for work privacy conflict in our sample is relatively low (45.8 vs. 67.7), which could be explained by the fact that the current sample is on average older (42.0 years) compared to the study by Wagner et al. (36.1 years). In this context, age is negatively correlated with size of conflict in our study and is similar to other findings that suggest that older people show a reduced risk for work-privacy-conflict [42]. In addition, only 61.9% in our sample are female, compared to 82.9% in the Wagner et al. sample. It has been shown, that women exhibit greater levels of work-privacy (or work-live) conflict compared to men due to the double burden of occupation and family tasks such as care work [43, 42]. Another contributor may be the type of medical setting, since physicians working in hospitals exhibit greater levels of conflict between work and private life. Our sample consisted of physicians working in outpatient as well as impatient care, whereas the sample by Wagner et al. only focussed on physicians working in hospitals, therefore the size of conflict may be greater. Apart from that, our results go align with an international validation study of COPSOQ III, obtaining a mean of 42 (range: 35 -51) across more than 23,000 employees [44].

One possible limitation could be found within the sample itself. Physicians with more burnout or stress due to time pressure (for instance because they are working part-time) may not have participated in this study. Additionally, the majority of participants in this study in working in areas with more than 500,000 inhabitants. Especially physicians, working in rural areas with less than 5,000 inhabitants (about 6.0%) are underrepresented. Another limitation was that it was not possible to differentiate between medical specialties. It may be interesting to consider this variable in future studies and investigate whether job size differently effects burnout and work-privacy conflict depending on the type of medical background. As this is a cross-sectional study, it is not possible to infer causality between the variables under investigation. Follow-up studies are needed to make comparisons that allow for statistical inferences and comprehensive conclusions.

In conclusion, the results of this study suggest that less medical attendance (i.e. reduced jobs size and part-time work) does not automatically reduce work-related stress, replicating and emphasizing similar findings [12]. However, with regard to work-privacy conflict, part-time work seems to improve overall working conditions, and allows for recovery and time for private matters. In this context, being younger and female does seem to play an important role and possible working hour-related interventions should be individualized to suit career and life phases that are characterized by specific needs (e.g. health care or professional training). Therefore, offering part-time contracts to physicians may be a way to keep as many as possible within the health care system and prevent physician shortage. This is the first study that investigated burnout and work-privacy conflict in a large sample of physicians with different working hour schedules. It builds up on previous studies, and underlines the impact of hours worked per week on occupational health of this medical profession. Due to its heterogeneity (for instance including physicians working in outpatient and inpatient care) it sheds light on the fact that these impacts may not equally account for all physicians. Besides considering different medical settings within analysis, future research could explore the influence of different medical specialities in order to complete the picture. Moreover, interventions should take into account specific job-related characteristics that may reduce workload and the risk for burnout, providing possibilities not only for physician’s recovery from work, but also for the healthcare system to recover from physician shortage without becoming a “chronic wound”. So far, work-family research has developed several innovative workplace interventions, such as schedule control, supervisor social support or on-site childcare possibilities (e.g. [45]) that could help to reduce work family conflict and reduce the risk to develop burnout. The question remains how these can be transferred to the health care sector, improving physician’s wellbeing.

## Data Availability

The datasets generated during and analyzed during the current study are not publicly available due to legal and ethical restrictions (participants of this study did not agree for their data to be shared publicly) but are available from the corresponding author on reasonable request.
